# The clinical manifestation, survival outcome and predictive prognostic factors of 137 patients with primary gastrointestinal lymphoma (PGIL): Strobe compliant

**DOI:** 10.1097/MD.0000000000009583

**Published:** 2018-01-05

**Authors:** Zhan Shi, Hao Ding, Qian Wen Shen, Xin Gang Lu, Jia Yan Chen, Xi Chen, Xi Tang

**Affiliations:** a Department of Medical Oncology; b Department of General Surgery; c Department of Radiation Oncology; d Department of Traditional Chinese Medicine of Hua’dong Hospital Affiliated to Fu’dan University, Shanghai, People's Republic of China.

**Keywords:** cytokine therapy, lymphoma and Hodgkin disease, primary gastrointestinal lymphomas, prognostication

## Abstract

This retrospective study aimed to investigate clinical characteristics and prognostic factors in patients with primary gastrointestinal lymphoma (PGIL) of Chinese population.

From January 2001 to December 2015, 137 patients diagnosed with PGIL were recruited. The clinical features, treatment, and follow-up information were analysed.

The median patient age was 62.3 years. With 18.47 months follow-up, the 2-year progress-free survival and overall survival rate was 74.9% and 75.5%, respectively. The overall response rate was 33.6%. Age≥60 years, advanced Lugano staging (≥stage IIE), elevated lactate dehydrogenase (LDH) levels, ≥2 extra-nodal involved sites, National Comprehensive Cancer Network International Prognostic Index (NCCN-IPI)≥4, Ki-67≥50% were associated with worse prognosis in univariate analysis (*P* < .05). By multivariate analyses, we determined that the involvement of extra-nodal involved sites was the only statistically significant poor prognostic factor in PGIL.

Age, staging, LDH levels, NCCN-IPI, Ki-67 especially involvement of multiple extra-nodal sites were associated with poor overall survival of PGIL.

## 1 Introduction

Lymphoma is a heterogeneous malignancy, showing a highly variable outcome especially for the primary gastrointestinal lymphoma (PGIL). Dawson's criteria are used for labeling primary gastrointestinal lymphoma that include (1) the absence of peripheral lymphadenopathy at the time of presentation; (2) lack of enlarged mediastinal lymph nodes; (3) normal total and differential white blood cell count; (4) predominance of bowel lesion at the time of laparotomy with only lymph nodes obviously affected in the immediate vicinity; and (5) no lymphomatous involvement of liver and spleen.^[1]^

PGIL representing the most common histological subtype of extra-nodal non-Hodgkin lymphoma (NHL) is usually not clinically specific and indistinguishable from other benign and malignant conditions.^[2]^ The etiology of PGIL is still not clear, autoimmune diseases, some immunosuppressive agents, *Helicobacter pylori* infection, high protein and high fat diet, environmental pollution are associated with increased incidence.^[3]^ The prognostic characterization of these patients is an essential prerequisite for the optimal risk-based therapeutic choice. Therefore, we investigated to identify the most prognostic and predictive index, not only to identify patients for more aggressive first-line treatments, but also to accurately select patients for clinical trials from which they will most benefit.

Given the location of the gastrointestinal (GI) tract and GI lymphoma association with infections such as *Helicobacter pylori* (HP) infection, celiac disease, inflammatory bowel disease, and autoimmune diseases, we consider PGIL as a distinct disease since their evaluation, diagnosis; management and prognosis are different from gastrointestinal cancer or NHL of lymph node origin. As we known, the International Prognostic Index (IPI) was used to play a role in predicting long-term survival for patients with NHL for decades,^[4]^ nowadays as a novel prognostic model, the National Comprehensive Cancer Network International Prognostic Index (NCCN-IPI) emphasizing age and lactate dehydrogenase (LDH) as the poor prognostic factors led to an unequivocal improvement in predicting survival of NHL patients.^[5]^ So far, it was difficult to find an ideal prognostic model without controversy to predict outcomes for patients with PGIL. Whether these currently available poor prognostic factors of NHL have the same suggestive impact on predicting survival outcome in the PGIL remains controversial. Thus, our study here is focused on the clinical characteristics and prognostic factors in patients with PGIL.

## 2 Materials and methods

### 2.1 Patients

The patients of this retrospective study were selected from those who were newly diagnosed by gastrointestinal endoscope biopsy or surgical pathology between December 2001 and December 2015 in Shanghai Hua’Dong Hospital, PRC. Meanwhile, patients who had a history of other malignant diseases or primary central nervous system involvement were excluded from the trial. The ethical committee approval was waived because this research is retrospective. The clinical features and follow-up information including details of history, physical examination, blood tests, staging, treatment, and outcome were collected from medical records.

Bulky disease was defined by the presence of one of the following 2 findings: (1) an abdominal node or nodal mass with a largest dimension of ≥7.5 cm as determined by an imaging study or (2) a mediastinal mass with a maximum width equal to or greater than one-third of the internal transverse diameter of the thorax at the T5/6 level as determined by a imaging study. The performance status was evaluated according to the Eastern Cooperative Oncology Group (ECOG) scale.^[6]^ The pathological specimens were obtained from endoscopic biopsies or surgical resections.

Staging and diagnostic procedures were conducted according to the Lugano staging system,^[7]^ which is modified from the Ann Arbor criteria for primary gastrointestinal non-Hodgkin's lymphoma. The different procedures employed for the pretreatment staging include endoscopic ultrasound (EUS), endoscopic biopsies, computed tomography (CT), magnetic resonance imaging (MRI), 18F-fluorodeoxyglucose positron emission tomography (FDG-PET), or molecular markers.^[8]^ Contrast-enhanced techniques and functional imaging such as perfusion CT can also help the monitoring, assessment, and prediction of response.^[9]^

Tumor response was classified according to the Resist 1.1 by National Cancer Institute-sponsored Working Group guidelines.^[10]^ Complete remission (CR) was defined as the complete disappearance of all detectable clinical evidence of disease and disease-related symptoms if these were present before therapy. Partial remission (PR) was defined as a ≥50% decrease in the sum of the product of the diameters of up to 6 of the largest dominant nodes or nodal masses. Stable disease (SD) was defined as a case that failed to attain the criteria needed for CR or PR but did not fulfil those for progressive disease (PD). Each patient was re-evaluated every 2 to 3 months for the first 2 years after treatment and every 6 months thereafter.

Overall survival (OS) was calculated from the start of diagnosis to the date of death or the last follow-up at which the patient was known to be alive. Two-year survival rate was used as the major clinical outcome. Progress-free survival (PFS) was defined as the time interval from the date of diagnosis to the date of lymphoma progression, clinical relapse from CR, or death as a result of any cause. Regular outpatient visit was the first choice and follow-up information was updated until December 2016.

### 2.2 Prognostic indices of IPI and NCCN-IPI

The IPI index, which is comprised of 5 clinical parameters, retains its validity in predicting long-term survival in patients with aggressive B-cell lymphoma especially for diffuse large B-cell lymphoma (DLBCL) for decades.^[4,11–13]^ However, IPI prognosis was not significantly associated with overall response rate (ORR). Recently, clinical data from the National Comprehensive Cancer Network (NCCN) member institutions demonstrated that NCCN-IPI, as a novel prognostic model, has been proposed with better discrimination of 5-year OS and PFS for patients with DLBCL treated with rituximab-containing chemotherapy as compared to the IPI for risk stratification.^[5]^ The standard for evaluation and risk stratification of IPI and NCCN-IPI are listed in Table 1.^[4]^

**Table 1:**
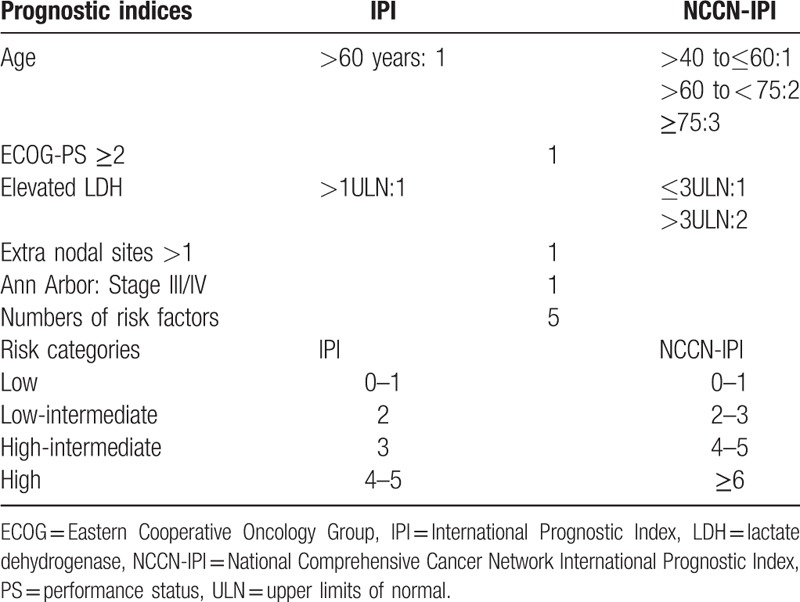
Prognostic indices and the risk stratification of IPI and NCCN-IPI.

### 2.3 Statistical analysis

Survival curves were plotted using the Kaplan–Meier method and the confidence intervals were calculated using the standard error. The log-rank test was performed to test the statistical significance. The prognostic value of different variables for survival outcome was estimated by multivariate analysis using the Cox regression model with the backward stepwise method. Hazard ratio (HR) with 95% confidence interval (CI) was calculated. The differences in survival among the groups with respect to variables were analyzed with the log-rank test. The *P*-values reported were 2-sided; a *P*-value of .05 or less was considered statistically significant. The potential prognostic factors were included in the Cox multiple regression model as *P* < .05 in the univariate analysis in order to decide which factor could be the independent prognostic factor for survival. All statistical analyses were performed with the Statistical Package for the Social Sciences (SPSS) (version 14.0 software, Microsoft).

## 3 Results

### 3.1 Patient clinical and histological characteristics

From December 2001 to December 2015, 137 gastrointestinal lymphoma patients were enrolled in this trial and no one was lost. The clinical and histological features of patient at the time of diagnosis are summarized in Table 2. The median age of the patients was 62.3 years (range, 19–93 years) with similar distribution of both sexes (68 males and 69 females; ratio = 1.0). The most three common sites of extra-nodal metastasis in our trial were spleen, bone, and liver. Around ≥2 extra-nodal invasion was present in 48% (n = 67) of patients. After chi-square test, there was no statistically significant correlation between Ki-67 high (>70%) and multiple extra-nodal sites. (*P* = .059).

**Table 2:**
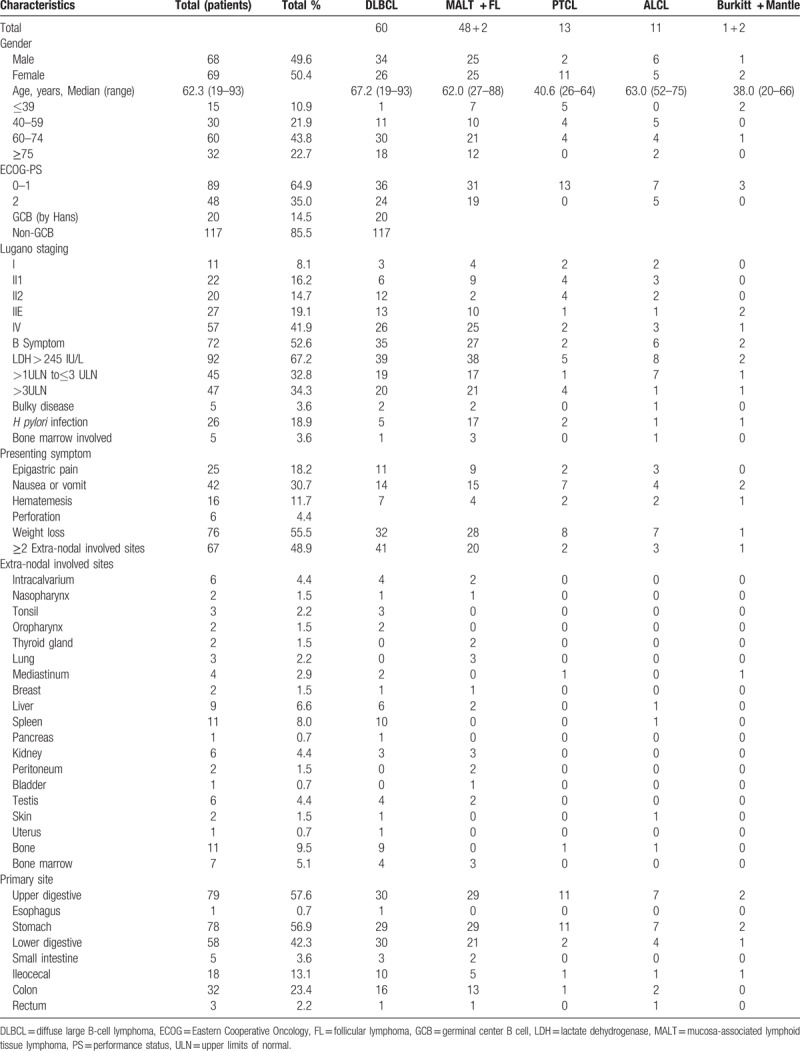
Clinical and histological characteristics of patients at diagnosis.

In our trial, the common primary sites of gastrointestinal lymphoma were the stomach (56.9%), followed by colon (23.4%), ileocecal region (13.1%), small intestine (3.6%), rectum (2.2%), and esophagus (0.7%), respectively. There were 79 cases in the upper digestive tract and 58 cases in the lower digestive tract. The clinical and histological characteristics of upper or lower digestive tract are listed in Table 3. As for the primary lymphoma of the lower digestive tract, the most common site of extra-nodal metastasis were spleen (differ from the upper digestive tract). As for the different primary site, there showed no significant difference in the clinical features including gender, age, Lugano staging, bulky disease, HP infection, extra-nodal involved sites, Ki-67 index, and even the proportion of histologic subtypes between the upper and lower digestive tract. Table 4 shows the clinical characteristics of PGIL with multiple extra-nodal involved sites. After analyzing different histological types of PGIL with multiple extra-nodal invasion in this trial, there were still no obvious differences in their clinical features except for *H pylori* infection, the number of MALT patients was significantly higher than that of DLBCL patients.

**Table 3:**
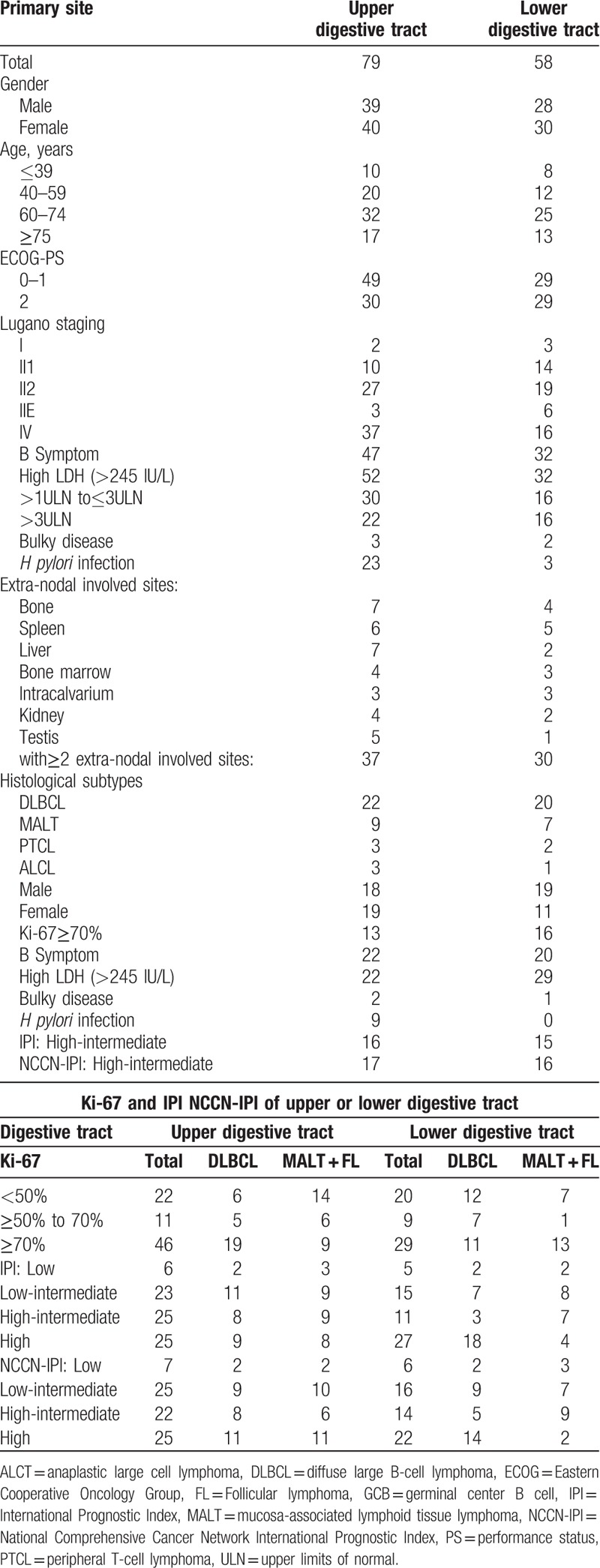
Clinical and histological characteristics of upper or lower digestive tract.

**Table 4:**
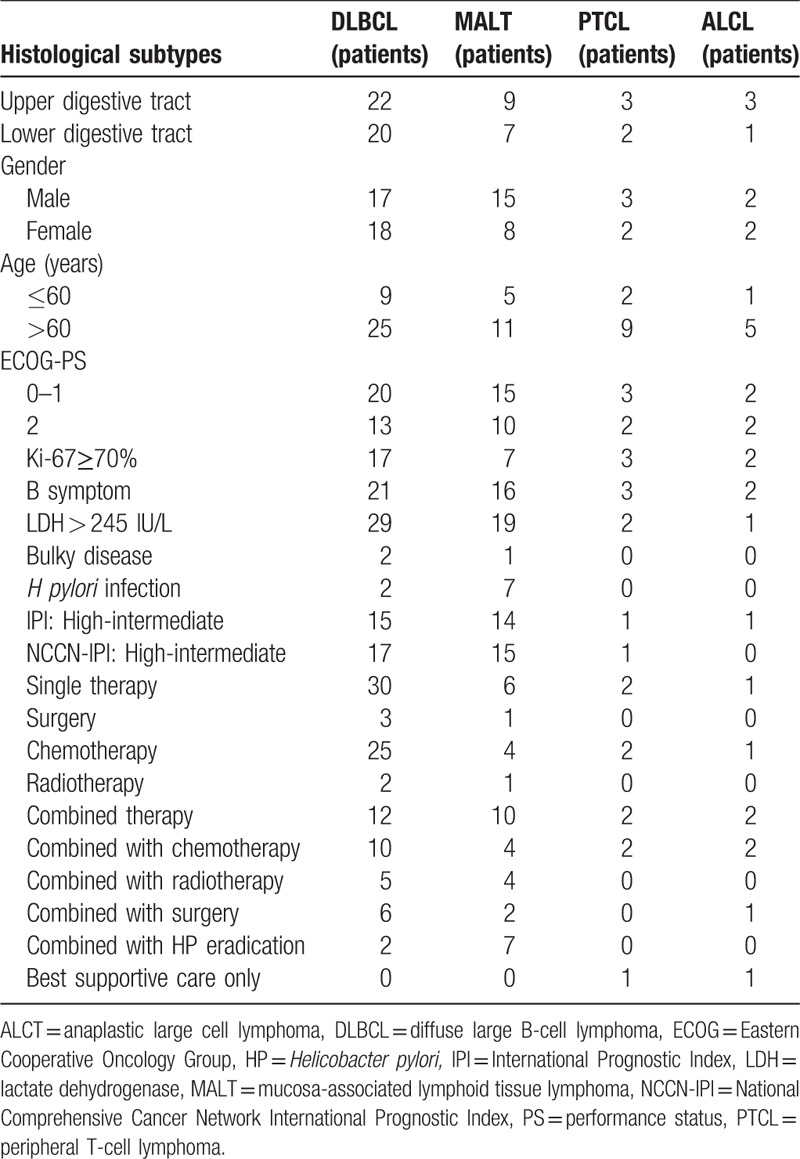
Clinical characteristics of PGIL with multiple extra-nodal involved sites.

The most predominant histological subtype in our trial is diffuse large B-cell lymphoma (DLBCL) (43.8%) followed by mucosa-associated lymphoid tissue lymphoma (MALT) (35.0%), both in the upper and lower digestive tract, just as the other research reported.^[2,14,15]^

The most common presenting symptoms were weight loss (55.5%), nausea or vomit (30.7%), epigastric pain (18.2%), and hematemesis (11.7%).

### 3.2 The risk groups of each prognostic model by IPI and NCCN-IPI

At diagnosis, the IPI and NCCN-IPI were available for 137 patients. The numbers and percentages of patients classified into the risk groups of each prognostic model (IPI and NCCN-IPI) are documented in Table 5. The majority of cases were in the high-intermediate and high risk group both in upper and lower digestive tract.

**Table 5:**
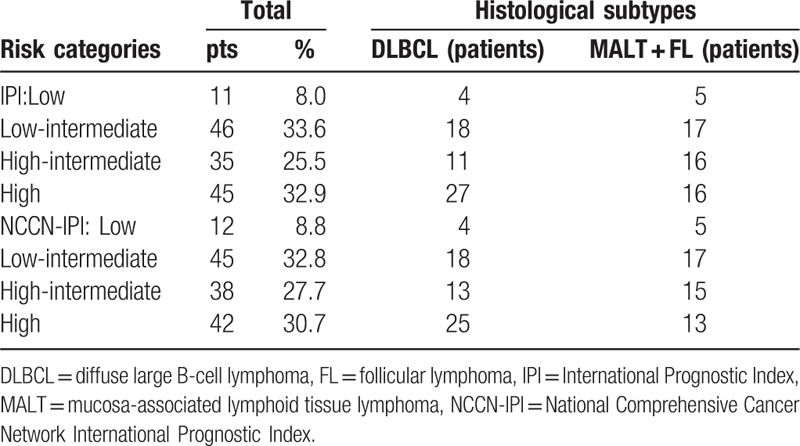
The risk groups of each prognostic model by IPI and NCCN-IPI.

### 3.3 First-line treatment and response to the first-line treatment

#### 3.3.1 First-line treatment

**Figure 1:**
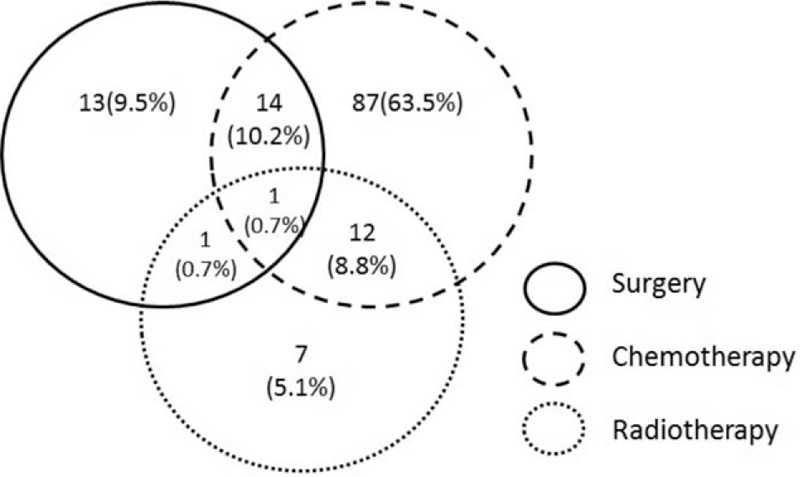
First-line treatment modalities.

Table 6 and Figure 1 showed that 107 people patients treated with single therapy, 28 with combination therapy, and the other 2 were treated with the best supportive care only. Among the 114 patients (83.2%) who received chemotherapy, 87 patients (63.5%) received only chemotherapy and 27 patients (19.7%) received chemotherapy combined with other treatment modalities. A total of 109 patients received at least 4 cycles of a standard-dose R-CHOP (rituximab, cyclophosphamide, doxorubicin, vincristine, and prednisone)-like regimen. Of these, 98 patients received chemotherapy for more than 6 cycles. While the median age of the patients was 62.3 years in this study, 11 patients did not complete 6 cycles of chemotherapy because they were unable to tolerate the adverse effects of chemotherapy and terminated chemotherapy or progression of the disease requiring second-line chemotherapy. The R-chop-like regimen with rituximab ranged from 1 to 8 cycles, with a median of 6.2 cycles. Other regimens included FC (fludarabine and cyclophosphamide) and hyper-CVAD (cyclophosphamide, vincristine, doxorubicin, dexamethasone, methotrexate, and cytarabine).

**Table 6:**
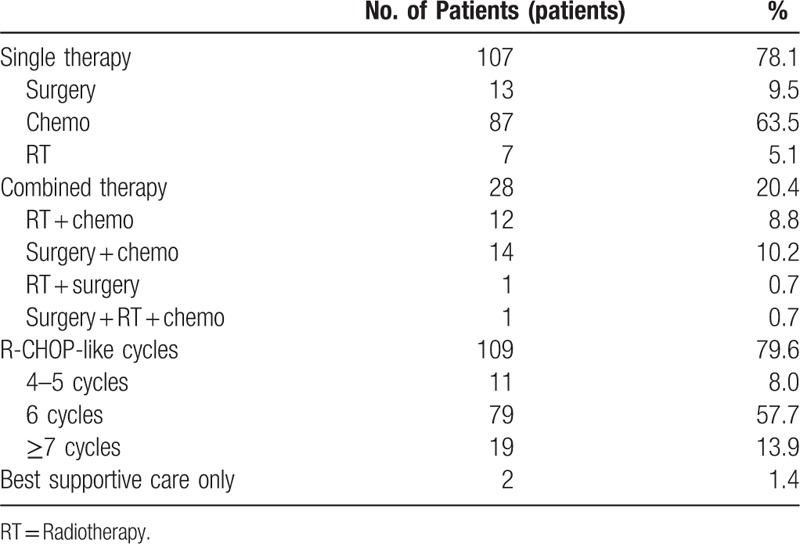
First-line Treatment modalities.

Surgical treatment either combined or as single therapy was performed in 29 patients (21.2%) because of perforation, intestinal obstruction, or gastrointestinal haemorrhage. The primary sites where surgery was conducted were stomach, small intestine, ileocecal region, and colon. The most common types of surgical treatment procedure were gastrectomy and gastrojejunostomy. Gastrojejunostomy was performed as palliative care in 3 patients with pyloric obstruction. None of these patients had preoperative chemotherapy or radiotherapy. In this study, 13 patients received surgical treatment alone in the first-line therapy, the reasons are as following: (1) This study spans from December 2001 to December 2015, before 2010 the treatment in Shanghai Hua’Dong Hospital, PRC for indolent lymphoma especially MALT patients was relatively conservative. Especially for the primary gastric MALT patients, they would be advised to taking follow-up after the surgery without further treatment. (2) Some patients suffered from severe adverse reactions after surgical treatment; resulting in delayed or discontinued further treatment. As shown in Figure 1, there was 1 case of patient with the surgery combined with radiotherapy and chemotherapy, the patient was a case of primary small intestine DLBCL lymphoma with bulky mass. Because of the poor curative effect of first-line chemotherapy, radiation therapy was performed, the tumor rupture occurred during the radiotherapy, and then intestinal repair surgery was given after the intestinal perforation. All treatments were suspended after operation, resulting in poor prognosis. The PFS of this case: 5.7 m and OS: 6.5 m. One patient, who underwent surgery combined with radiotherapy, was a patient with early primary gastric MALT who had gastric perforation during the radiotherapy of the stomach and underwent gastric repair surgery after. The PFS of this case: 15.7 m and OS: 18.9 m.

Of the 21 patients (15.3%) who received radiotherapy as single or combined therapy. There were 7 cases with single radiotherapy in this study that were all primary gastric MALT lymphoma. After gastroscopy and ultrasonic gastroscopy, these 7 primary gastric MALT lesions were confirmed as infiltrating into the serous layer of the stomach without invasion of the entire layer of the stomach or lymph node metastasis and distant organ metastasis. Therefore the 7 early stage primary gastric MALT cases just conducted alone with the stomach radiotherapy and then follow up. In this study, there were 29 patients with primary gastric DLBCL. Of 29 patients, 7 patients who received consolidation gastric or lesion radiotherapy followed by achieving CR after first-line chemotherapy or less-satisfied with tumor regression. Therefore, 7 patients were treated with chemotherapy combined with radiotherapy. In addition, 5 patients with large masses of bulky were included in the mediastinum, the abdominal cavity and the pelvic cavity respectively. These 5 bulky cases were treated with radiation after the first-line chemotherapy. The radiotherapy dosage is about 36 to 40 Gray (Gy) and 2 Gy/f. While 7 patients with early stage primary gastric MALT lymphoma received anti-HP treatment and consolidation radiotherapy which dosage was about 30.6 Gray (Gy) and 1.8 Gy/f.

*H pylori* (+) MALT lymphomas were regularly treated with *H pylori* eradication therapy.^[15]^ Around 26 patients (18.9%) underwent treatment for *H pylori* eradication as they were diagnosed with *H pylori* (+) MALT lymphomas. Of these 26 patients, 46.2% were DLBCL, and 53.8% were MALT. Especially for the 7 primary gastric MALT patients which infiltrated only to the mucosal layer, they would be advised to taking anti HP treatment alone as soon as diagnosed or began after the gastric radiotherapy and then follow-up. When it comes to the HP therapy combined with chemotherapy, as we know standard anti HP triple therapy requires combination of PPI and 2 antibiotics for 10 to 14 days, and meantime PPI medications should be discontinued for more than 2 weeks before treatment. In view of the fact that chemotherapy cannot do without the protective effect of PPI on stomach, the other 19 HP positive patients were treated with anti-HP therapy during the follow-up period after completion of the first-line intravenous chemotherapy.

#### 3.3.2 Recent response to the first-line treatment

The recent response to the first-line treatment is shown in Tables 7 and 8. Notably, 46 out of 137 patients who had achieved ORR at the end of the first-line treatment, therefore the ORR was 31.6%. Otherwise, 34 (24.8%) patients had reached PD in the follow-up. As aggressive lymphoma, the ORR rate of DLBCL (40.0%) is slightly higher than that of indolent lymphoma MALToma + FL (32.0%).

**Table 7:**
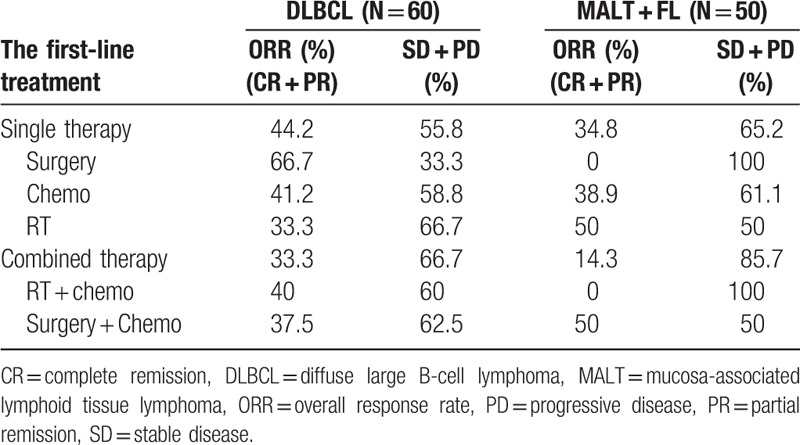
The response evaluation to the first-line treatment of DLBCL, MALT + FL.

**Table 8:**
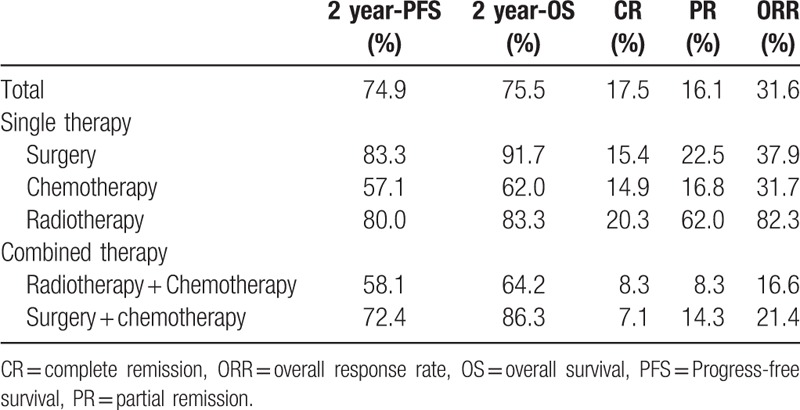
Responses to the first-line single treatment and survival outcomes.

#### 3.3.3 Survival and treatment outcome

Till the last follow-up in December 2016, the median durations of follow-up after diagnosis was 18.47 months (range, 3.43–133.9 months) and the follow-up rate was 100%. During the follow-up period, 34 patients relapsed and 21 deaths reported eventually. A total of 15 patients died of pneumonia or severe septic shock, 2 died of lymphoma relapse or hemophagocytic syndrome, 1 died of complete intestinal obstruction, 2 died of cerebrovascular events, and 1 died of secondary pancreatitis.

**Figure 2:**
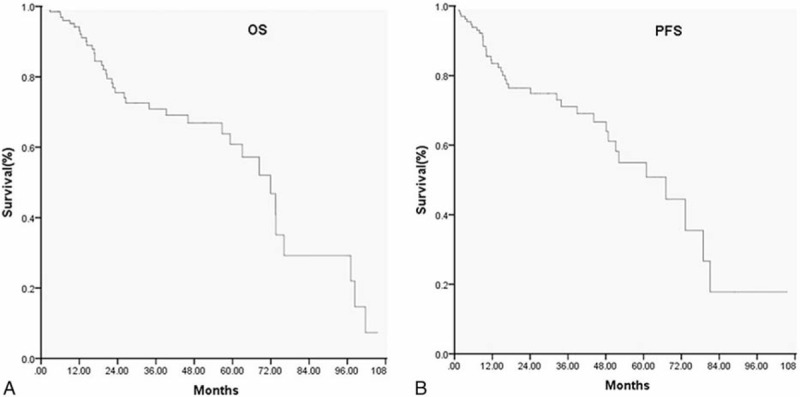
(A) Overall survival (OS), (B) progression-free survival (PFS) for all patients. OS = overall survival, PPFS = progress-free survival.

The 2-year PFS, OS rate, which were estimated by using the Kaplan–Meier method, was 74.9% ± 4.4%, and 75.5% ± 4.6%, respectively (Fig. 2), with mean survival time (MST) of 62.4 months (95% CI 53.7–71.0). The 2-year OS rate for upper digestive tract and lower digestive tract were 77.0% ± 5.8%, and 74.0% ± 7.2%, respectively. These data above suggested that primary lymphoma of the upper digestive tract may have a better prognosis than the lower gastrointestinal tract.

#### 3.3.4 Survival analyses among Prognostic factors

**Figure 3:**
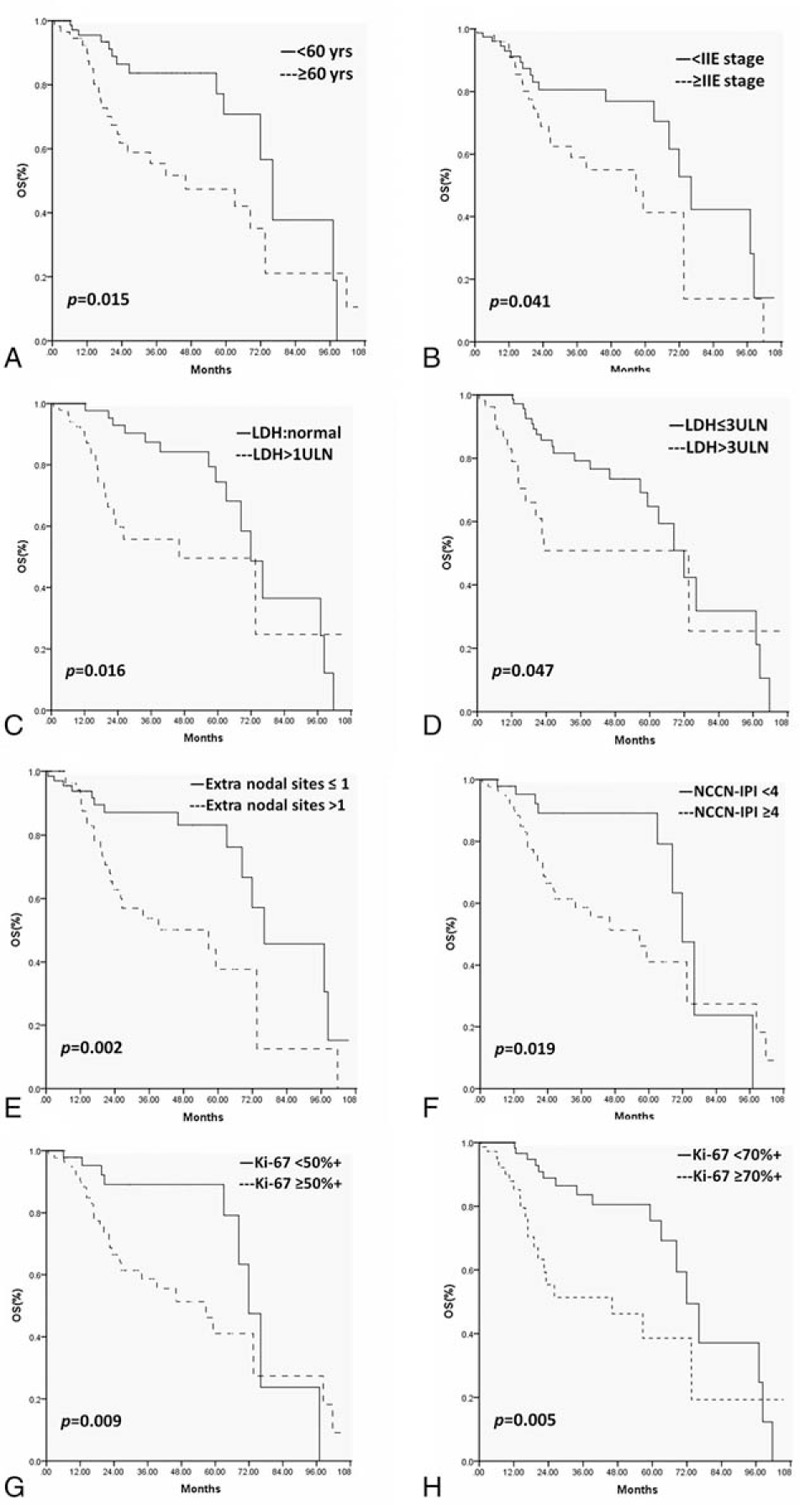
(A–H) Univariate analyses for overall survival (OS), (A) age ≥ 60 years, (B) advanced Lugano staging (≥stage IIE), (C) elevated LDH levels (>245 U/L), (D) LDH ≥ 3ULN, (E) ≥2 extra-nodal involved sites, (F) NCCN-IPI ≥ 4 (high-intermediate risk group), (G) Ki-67 (by IHC) ≥50%+, and (H) Ki-67 (by IHC) ≥70%+. IHC = immunohistochemistry, LDH = lactate dehydrogenase, NCCN-IPI = National Comprehensive Cancer Network International Prognostic Index, OS = overall survival, ULN = upper limits of normal.

**Figure 4:**
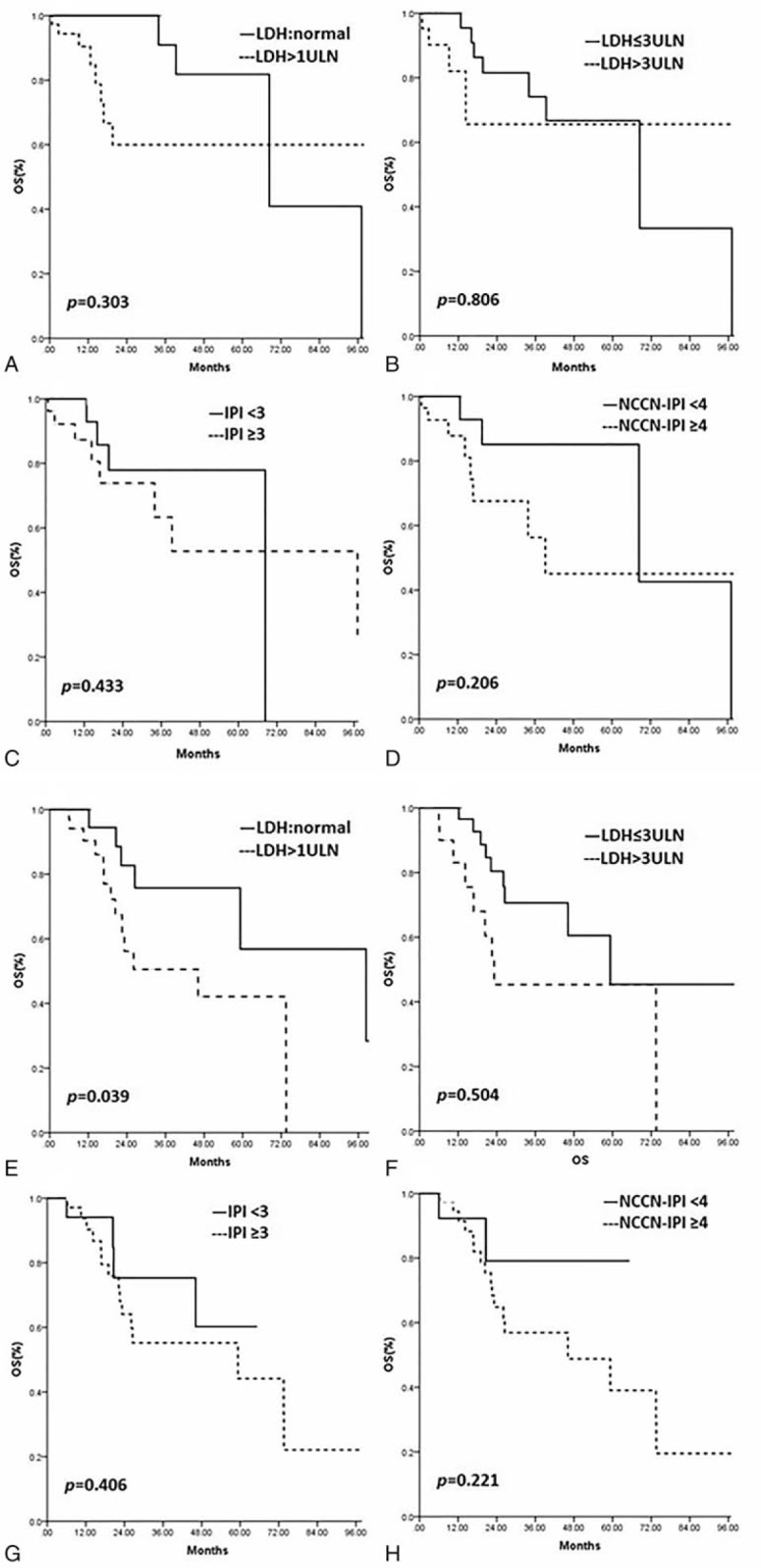
(A–D) Univariate analyses for overall survival (OS) in MALT of PGIL. (A) Elevated LDH levels (>245 U/L), (B) LDH ≥ 3ULN, (C) IPI <3, (D) NCCN-IPI ≥ 4 (E–H). Univariate analyses for overall survival (OS) in DLBCL of PGIL. (E) Elevated LDH levels (>245 U/L), (F) LDH ≥ 3ULN, (G) IPI <3, (H) NCCN-IPI ≥ 4. DLBCL = diffuse large B-cell lymphoma, LDH = lactate dehydrogenase, MALT = mucosa-associated lymphoid tissue lymphoma, NCCN-IPI = National Comprehensive Cancer Network International Prognostic Index, OS = overall survival, PGIL = primary gastrointestinal lymphoma, ULN = upper limits of normal.

In univariate analysis among all potential prognostic factors: age ≥ 60 years, advanced Lugano staging (≥stage IIE), elevated LDH levels (>245 U/L), LDH ≥ 3ULN (upper limits of normal), ≥2 extra-nodal involved sites, NCCN-IPI ≥ 4 (high-intermediate and high-risk group), Ki-67 (by IHC) ≥50% + and Ki-67 (by IHC) ≥70% + were predictive poor prognostic factors for 2-year OS (*P* < .05). Kaplan–Meier analysis showed that there was no apparent prognostic significant correlation between other variables like age ≥ 70 years, ECOG-PS≥2, male, B symptom, IPI≥3, primary lymphoma location, and survival (Table 9) (Fig. 3). After analyzing the survival curve on IPI and NCCN-IPI and LDH in MALT and DLBCL of PGIL, we found that there was no apparent prognostic significant correlation between IPI and NCCN-IPI and LDH in MALT of PGIL and survival. But in DLBCL of PGIL, the results showed that LDH≥1ULN (*P* = .039) was the only statistically significant poor prognostic factor (Fig. 4).

**Table 9:**
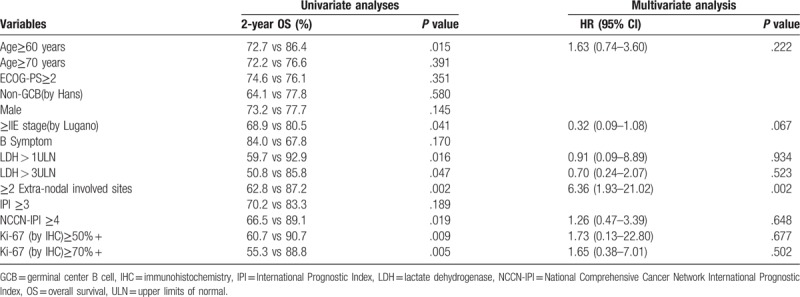
Univariate and multivariate analysis of prognostic factors for 2-year OS.

In multivariate analyses (MVA) by Cox regression analysis, the results showed that ≥2 extra-nodal involved sites (HR = 6.36, 95% CI: 1.93–21.02, *P* = .002) was the only statistically significant poor prognostic factor (Table 9).

## 4 Discussion

Primary gastrointestinal malignant lymphoma accounts for about 30% to 40% of all extranodal lymphomas, but only accounts for 1% to 8% of all gastrointestinal malignancies.^[2]^ Primary gastrointestinal lymphoma is relatively rare but an aggressive lymphoma that may arise de novo. It most commonly occurs in men with a median age of 50 to 60 years old.^[16]^ There has been a tremendous leap in the diagnosis, staging, and management of gastrointestinal lymphoma in the last 2 decades.

In this series, there were 137 patients with PGIL with similar distribution of both sexes, differ from the male predominance result corresponds to previous observations.^[17,18]^ Thus, although virtually lymphoma can arise from any region of the gastrointestinal tract, the stomach is the most common extra-nodal site of NHL presentation, representing 55% to 65% of all gastrointestinal lymphomas.^[19,20]^ Primary gastric lymphoma is often involved in multiple sites, and the involvement of gastric fundus and gastric body is the most common.^[21]^ Small intestinal lymphoma was present in the distal ileum. Primary colorectal lymphoma is rare, with most of the rectum and cecum, and large intestine lymphoma accounts for 10% of the digestive tract lymphoma, accounting for about 0.08% of the colorectal cancer. According to our results, the most commonly involved sites in term of its occurrence are stomach (56.9%) followed by colon (23.4%) and ileocecal region (13.1%) which corresponds to previous series.

Because of the low incidence of PGIL, the symptoms are not typical, so it is easy to cause missed diagnosis before serious complications occur. Thus, history of chronic gastritis patients usually have years of private prosecution, long-term medication treatment, without timely endoscopic examination, or who received multiple electronic gastro-scope, and endoscopic biopsy showed no signs of tumor specific. Weight loss, nausea or vomit, epigastric pain, hematemesis were the most common symptoms in our study, as reported in other series.^[22,23]^ The majority of patients with stage I and II by Lugano staging system were reported with 65% to 82%.^[24]^ In this trial, there were 80 cases (58.1%) of stages I and II with 57 cases (41.9%) of stage IV, similar to other literatures.^[22,23]^

According to World Health Organization (WHO) classification, the predominant histological subtypes of PGIL are marginal zone B-cell lymphoma of MALT and DLBCL.^[25]^ Histopathologically, most primary gastrointestinal lymphoma in our trial as are of B cell lineage with very few T-cell lymphomas just match previous researches.^[26]^ In this retrospective study, the proportions of the DLBCL (43.8%) and MALT lymphoma (35.1%) were similar to other research.^[17]^

Regarding treatment approach of PGIL, there are many options including surgical resection, HP eradication, chemotherapy, and radiotherapy. Traditionally, radical gastrectomy was regarded as the frontline treatment for PGIL. Actually, in recent years surgery has gradually been replaced by chemotherapy and radiotherapy which has been front-line treatment in Shanghai Hua’Dong Hospital, PRC since 2011 after the guideline for lymphoma published by Japanese Gastric Cancer Association (JGCA).^[27]^ However, surgery is now recommended as urgent treatment of patients presenting severe perforation or bleeding, and as palliative treatment.^[28,29]^ In this study, there were 114 patients (83.2%) received chemotherapy as single or combined therapy, 21 patients (15.3%) received radiotherapy. Meanwhile, 28 patients (20.4%) were treated with at least 2 different types of therapies which are common in the era of immunotherapy. Infection with *H pylori* appears to be a vital causal factor in the development of MALT lymphomas. Previous studies have showed that eradication of *H pylori* can lead to lymphoma regression. In this study, all 26 patients (18.5%) who underwent treatment for HP eradication were still alive. Our data suggested that conservative treatment modalities should be preferred in low-grade MALT lymphoma patients.

As literature report that the response rate of PGIL after first-line treatment was 83.3%. A retrospective analysis of 27 cases of primary intestinal lymphoma by Khosla et al^[30]^ showed that OS was up to 79.50% in the patients with surgery plus chemotherapy for the past 5 years, and was significantly better than that of the patients with chemotherapy alone. Studies showed that surgical resection of the lesion may improve quality of life, but not overall survival.^[31,32]^ In this study, patients received emergency surgery and mostly palliative surgery. Both single and combined with surgery were added to only 29 cases (21.2%). Therefore, taking into account the sample size is not balanced we regretted that we cannot analyse the surgery in predicting survival in PGIL. In our trial, the ORR was 31.6%. As aggressive lymphoma, the ORR rate of DLBCL (40.0%) is slightly higher than that of indolent lymphoma MALToma + FL (32.0%). The reason for this result may be that the sample size of this study was small or that the treatment of DLBCL was more active as aggressive lymphoma, and the treatment of MALT was conservative as indolent lymphoma. Moreover, the prognosis of primary stomach MALT was worse after the occurrence of tumor emergency and surgery. The early stage primary stomach MALT could only be evaluated by endoscopic ultrasonography, but the tumor regression was not obvious under ultrasound endoscopy, so the response evaluation could only be evaluated as stable.

In previous studies, there are multiple factors that contribute to survival of NHL including IPI, high-grade histology, poor performance status (PS), and surgical resection.^[18,19,33,34]^ While general prognosis of PGIL involved host-related clinical factors and tumor individual characteristics, such as age >60 years, poor PS, histological subtype, stage, elevated LDH, and so on.^[18,19,33–37]^ But it was still difficult to find an ideal prognostic model without controversy to predict outcomes for patients with PGIL. Based on our results, among all potential prognostic factors, age ≥ 60 years, advanced Lugano staging (≥stage IIE), elevated LDH levels (>245 U/L), LDH ≥ 3ULN, ≥2 extra-nodal involved sites, NCCN-IPI≥4 (high-intermediate and high-risk group), Ki-67 ≥50%+ and Ki-67 ≥70%+ were predictive poor prognostic factors for 2-year OS in univariate analysis (Table 8). Therefore, although our data revealed that there was no apparent prognostic significant correlation between IPI≥3 and NCCN-IPI≥4 and survival in multivariate analysis, the NCCN-IPI showed more powerful prognostic value than the IPI for patients with PGIL in univariate analysis. While LDH level was generally considered as a prognostic factor, and its level higher than the upper limit of the normal range implied poor prognosis.^[37,38]^ The result above was consistent with previous research.

Furthermore, based on multivariate analysis of 2-year-OS, only ≥2 extra-nodal involved sites retained their significance. The 2-year OS rate for upper digestive tract and lower digestive tract were 77.0% and 74.0%, respectively. These above data suggested that primary lymphoma of the upper digestive tract may have a better prognosis than the lower gastrointestinal tract. As a clinical parameter both in IPI and NCCN-IPI, involvement of multiple extra-nodal sites had a stronger predictive value for poor prognosis compared with the other predictive poor prognostic factors for 2-year OS in univariate analysis and multivariate analysis as well. Our results were different from some other studies suggesting that specific extra-nodal involved sites have more prognostic value for PGIL. The new knowledge from this study revealed that the involvement of multiple extra-nodal sites at diagnosis, among the 5 prognostic indices of IPI and NCCN-IPI as well, was the only statistically significant poor prognostic factor both in univariate analysis and multivariate analysis as well. This conclusion tells us that the involvement of organs other than gastrointestinal or lymph node maybe a critical prognostic factor for PGIL. In addition, the involvement of multiple organs outside the gastrointestinal tract and lymph node may indicate that the tumor has been transported to distant organs via blood flow, resulting in a poor therapeutic sensitivity and a significantly shorter survival time. Those patients of this group may need intensive or aggressive first-line chemotherapy to improve outcome.

However, the main limitation of this study was its retrospective design with a relatively small sample size. And the data come from a single center, so the results may not represent the Chinese population well. Moreover, the patients received both single and combined with surgery and radiotherapy were only 29 and 21 cases, respectively, in this study (Fig. 1 and Table 6). Therefore, taking into account the sample size is not balanced, we regretted that we cannot analysis the surgery and radiotherapy in predicting survival in PGIL. Further randomized prospective studies with a large sample size are needed to guide the optimal management for patients with PGIL.

## 5 Conclusions

Taken together, our data revealed that age ≥ 60 years, Lugano staging ≥IIE, LDH levels >245U/L, NCCN-IPI≥4, Ki-67 especially involvement of multiple extra-nodal sites at diagnosis had a prognostic value in predicting survival in PGIL. Those patients with the involvement of multiple extra-nodal sites may need intensive or aggressive first-line chemotherapy to improve outcome. Thus, additional large prospective studies are warranted.
